# Supplementary material for: Fruit and Vegetable Concentrate Supplementation and Cardiovascular Health: A Systematic Review from a Public Health Perspective
Source: J Clin Med. 2019 Nov 8;8(11):1914. doi: 10.3390/jcm8111914 (PMC6912365; doi:10.3390/jcm8111914)
Supplement: Supplementary file 1 [file jcm-08-01914-s001.pdf]

Table S1. Search strings and retrieved records. Numbers reported in bold characters are the final references included. Numbers reflect the records that were retrieved until June 22nd, 2018.

**Search string**	**N of records**
PubMed	
fruits[MeSH Terms] OR "fruit" OR "fruits"	146515
vegetables[MeSH Terms] OR "vegetable" OR "vegetables"	65817
"concentrate" OR "concentrates"	42962
((fruits[MeSH Terms] OR "fruit" OR "fruits") OR (vegetables[MeSH Terms] OR "vegetable" OR "vegetables")) AND ("concentrate" OR "concentrates")	1285
((fruits[MeSH Terms] OR "fruit" OR "fruits") OR (vegetables[MeSH Terms] OR "vegetable" OR "vegetables")) AND ("concentrate" OR "concentrates")	**310**
Scopus	
TITLE-ABS-KEY(fruit) OR TITLE-ABS-KEY(fruits)	280395
TITLE-ABS-KEY(vegetable) OR TITLE-ABS-KEY(vegetables)	196186
ALL(concentrate) OR ALL(concentrates)	292282
((TITLE-ABS-KEY(fruit) OR TITLE-ABS-KEY(fruits)) OR (TITLE-ABS-KEY(vegetable) OR TITLE-ABS-KEY(vegetables))) AND (ALL(concentrate) OR ALL(concentrates))	9970
((TITLE-ABS-KEY(fruit) OR TITLE-ABS-KEY(fruits)) OR (TITLE-ABS-KEY(vegetable) OR TITLE-ABS-KEY(vegetables))) AND (ALL(concentrate) OR ALL(concentrates)) AND (LIMIT-TO(EXACTKEYWORD, "Human") AND LIMIT-TO(SUBJAREA, "MEDI"))	**1287**
Web of Science	
TOPIC: (fruit) OR TOPIC: (fruits)	220306
TOPIC: (vegetable) OR TOPIC: (vegetables)	102998
TOPIC: (concentrate) OR TOPIC: (concentrates)	276583
((TOPIC: (fruit) OR TOPIC: (fruits)) OR (TOPIC: (vegetable) OR TOPIC: (vegetables))) AND (TOPIC: (concentrate) OR TOPIC: (concentrates))	3959
((TOPIC: (fruit) OR TOPIC: (fruits)) OR (TOPIC: (vegetable) OR TOPIC: (vegetables))) AND (TOPIC: (concentrate) OR TOPIC: (concentrates))	**232**
Embase	
'fruit'/exp OR 'fruit'	165,734
'vegetable'/exp OR 'vegetable'	204,332
'concentrate' OR 'concentrates'	67,055
(('fruit'/exp OR 'fruit') OR ('vegetable'/exp OR 'vegetable')) AND ('concentrate' OR 'concentrates')	2,372
(('fruit'/exp OR 'fruit') OR ('vegetable'/exp OR 'vegetable')) AND ('concentrate' OR 'concentrates')	**549**

Table S2. Articles used to estimate the effect size of a single concentrate product on a specific clinical surrogate.

**Population**	**Healthy**						**Metabolic syndrome**						**Hypertension**					
**Surrogate**	TC	LDL	HCY	BMI	SBP	TNF-α	TC	LDL	HCY	BMI	SBP	TNF-α	TC	LDL	HCY	BMI	SBP	TNF-α
Encapsulated FV concentrate	Novembrino 2011 [1]	Plotnick 2003 [2]	Panunzio 2003 [3]	-	-	Lamprecht 2007 [4]	Ali 2011 [5] Williams 2017 [6]*	Ali 2011 [5] Williams 2017 [6]*	-	Ali 2011 [5] Williams 2017 [6]*	-	Lamprecht 2013 [7] Williams 2017 [6]*	-	-	-	-	-	-
Artichoke	-	-	-	-	-	-	-	-	-	-	-	-	Roghani 2009 [8]	Roghani 2009 [8]	-	Roghani 2009 [8]	Roghani 2009 [8]	-
Jerusalem artichoke	-	-	-	-	-	-	Antal 2008 [9]	Antal 2008 [9]	-	-	-	-	-	-	-	-	-	-
Orange juice	Cesar 2010 [10]	Cesar 2010 [10]	-	Cesar 2010 [10]	-	-	Cesar 2010 [10]	Cesar 2010 [10]	-	Cesar 2010 [10]	-	-	-	-	-	-	-	-
Fruit and vegetable drink	George 2012 [11]	George 2012 [11]	George 2012 [11]	George 2012 [11]	George 2012 [11]	-	-	-	-	-	-	-	-	-	-	-	-	-
Cherry Juice	Lynn 2014 [12]	-	-	-	Lynn 2014 [12]	-	-	-	-	-	-	-	-	-	-	-	-	-
Fruit juice	Van Den Berg 2001 [13]	Van Den Berg 2001 [13]	-	-	Van Den Berg 2001 [13]	-	-	-	-	-	-	-	-	-	-	-	-	-

*The pooled effect was used for the purpose of the simulation

Table S3. Composition of the concentrates administered in each study. The table reports the most frequently presented composition data in the papers included in the analysis. The empty cells indicate that the information was not reported in the paper.

		Type	Protein	Carbohydrate	Fat	Fibre	Beta-carotene	Vitamin C	Vitamin E	Folate	Calcium	Total sugar	Energy	Oligofructose
Ali et al. 2011 [5]	6 capsules	Encapsulated FV concentrate and berry juice					7.5 mg	276 mg	72 mg (RRR-alpha-Tocopherol)	780 µg	80 mg			
	4 capsules	Encapsulated FV concentrate					7.5 mg	234 mg	30 mg (RRR-alpha-Tocopherol)	420 µg	60 mg			
Antal et al. 2008 [9]		Jerusalem artichoke												14 g/day
Cesar et al. 2010 [10]	750 mL	Orange juice						258 mg		135 µg		64 g	1318 KJ	
George et al. 2012 [11]	100 mL	Fruit and Vegetable-1	1 g	13.6 g	0.5 g	1.5 g	2.6 mg	36 g					263 KJ	
	100 mL	Fruit and Vegetable-2	2 g	17 g	0.4 g	1.5 g	3.3 mg	48.5 g					334 KJ	
Lamprecht et al. 2007 [4]	6 capsules	Encapsulated FV concentrate					7.5 mg	200 mg	60 mg (RRR-alpha-tocopherol)	600 µg			63 KJ/d	
Lamprecht et al. 2013 [7]	6 capsules	Encapsulated FV concentrate					7.5mg	200mg	60 mg (RRR-alpha-tocopherol)	600 µg			63 KJ/d	
Lynn et al. 2014 [12]	30 mL	Cherry juice	1.1 g	24.5 g	Traces	2.6 g	408 µg	9.72 mg					102 kcal	
Novembrino et al. 2011 [1]	4 capsules	Encapsulated FV concentrate					7.5 mg	234 mg	32 mg	420 µg			42 kJ/d	
Panunzio et al. 2003 [3]	2 capsules of fruit	Encapsulated FV concentrate						90 mg	15 mg					
	2 capsules of vegetables	Encapsulated FV concentrate						50 mg	15 mg					
Plotnick et al. 2003 [2]	2 capsules	Encapsulated FV concentrate					9.275 i.u.	20.4 mg	24.7mg					
Roghani-Dehkord et al. 2009 [8]	Nutrients contents reported in % (considering that the water content is 71.3%)	Artichoke Leaf Juice	5.60%	5.80%	1.20%	16.10%								

Van Den Berg et al. 2001 [13]	330 mL	Fruit juice	1.2 g	33.3 g	<0.2 g		<0.03 mg	52.5 mg					580 KJ	
Williams et al. 2017 [6]	6 capsules	Encapsulated FV concentrate					3.05 mg/day	300 mg/day		350 mg/day				

References

1. Novembrino, C. ;Cighetti, G. ;De Giuseppe, R. ;Vigna, L. ;de Liso, F. ;Pellegatta, M. ;Gregori, D. ;Maiavacca, R. ;Bamonti, F. Effects of encapsulated fruit and vegetable juice powder concentrates on oxidative status in heavy smokers. *J. Am. Coll. Nutr.* **2011**, *30*, 49–56.
2. Plotnick, G.D. ;Corretti, M.C. ;Vogel, R.A. ;Hesslink Jr, R. ;Wise, J.A. Effect of supplemental phytonutrients on impairment of the flow-mediated brachial artery vasoactivity after a single high-fat meal. *J. Am. Coll. Cardiol.* **2003**, *41*, 1744–1749.
3. Panunzio, M.F. ;Pisano, A. ;Antoniciello, A. ;Di Martino, V. ;Frisoli, L. ;Cipriani, V. ;Mongelli, M.A. ;Bronzetti, G. Supplementation with fruit and vegetable concentrate decreases plasma homocysteine levels in a dietary controlled trial. *Nutr. Res.* **2003**, *23*, 1221–1228.
4. Lamprecht, M. ;Oettl, K. ;Schwaberger, G. ;Hofmann, P. ;Greilberger, J.F. Several indicators of oxidative stress, immunity, and illness improved in trained men consuming an encapsulated juice powder concentrate for 28 weeks. *J. Nutr.* **2007**, *137*, 2737–2741.
5. Ali, A. ;Yazaki, Y. ;Njike, V.Y. ;Ma, Y. ;Katz, D.L. Effect of fruit and vegetable concentrates on endothelial function in metabolic syndrome: A randomized controlled trial. *Nutr. J.* **2011**, *10*.
6. Williams, E.J. ;Baines, K.J. ;Berthon, B.S. ;Wood, L.G. Effects of an encapsulated fruit and vegetable juice concentrate on obesity-induced systemic inflammation: A randomised controlled trial. *Nutrients* **2017**, *9*.
7. Lamprecht, M. ;Obermayer, G. ;Steinbauer, K. ;Cvirn, G. ;Hofmann, L. ;Ledinski, G. ;Greilberger, J.F. ;Hallstroem, S. Supplementation with a juice powder concentrate and exercise decrease oxidation and inflammation, and improve the microcirculation in obese women: randomised controlled trial data. *Br. J. Nutr.* **2013**, *110*, 1685–1695.
8. Roghani-Dehkordi, F. ;Kamkhah, A.F. Artichoke leaf juice contains antihypertensive effect in patients with mild hypertension. *J. Diet. Suppl.* **2009**, *6*, 328–341.
9. Antal, M.; Péter, S.; Regöly-Mérei, A.; Biró, L.; Arató, G.; Schmidt, J.; Nagy, K.; Greiner, E.; Lásztity, N.; Szabó, C. Effects of oligofructose containing diet in obese persons. *Clin. Exp. Med. J.* **2010**, *4*, 141–152.
10. Cesar, T.B.; A., N.P.;. Araujo, M.P.;. Vinagre, C.C.;. Maranhao, R.C. Orange juice decreases low-density lipoprotein cholesterol in hypercholesterolemic subjects and improves lipid transfer to high-density lipoprotein in normal and hypercholesterolemic subjects. *Nutr Res* **2010**, *30*, 689–94.
11. George, T.W. ;Paterson, E. ;Waroonphan, S. ;Gordon, M.H. ;Lovegrove, J.A. Effects of chronic consumption of fruit and vegetable puree-based drinks on vasodilation, plasma oxidative stability and antioxidant status. *J. Hum. Nutr. Diet.* **2012**, *25*, 477–487.
12. Lynn, A.; Mathew, S.; Moore, C.T.; Russell, J.; Robinson, E.; Soumpasi, V.; Barker, M.E. Effect of a Tart Cherry Juice Supplement on Arterial Stiffness and Inflammation in Healthy Adults: A Randomised Controlled Trial. *Plant Foods Hum. Nutr.* **2014**, *69*, 122–127.
13. Van Den Berg, R. ;Van V., T. ;Broekmans, W.M.R. ;Cnubben, N.H.P. ;Vaes, W.H.J. ;Roza, L. ;Haenen, G.R.M.M. ;Bast, A. ;Van Den Berg, H. A vegetable/fruit concentrate with high antioxidant capacity has no effect on biomarkers of antioxidant status in male smokers. *J. Nutr.* **2001**, *131*, 1714–1722.
